# Increased dermal collagen bundle alignment in systemic sclerosis is associated with a cell migration signature and role of *Arhgdib* in directed fibroblast migration on aligned ECMs

**DOI:** 10.1371/journal.pone.0180751

**Published:** 2017-06-29

**Authors:** Lizhi Cao, Robert Lafyatis, Linda C. Burkly

**Affiliations:** 1 Department of Neuroimmunology, Biogen, Cambridge, Massachusetts, United States of America; 2 Department of Medicine, University of Pittsburgh, Pittsburgh, Pennsylvania, United States of America; University of Bergen, NORWAY

## Abstract

Systemic sclerosis (SSc) is a devastating disease affecting the skin and internal organs. Dermal fibrosis manifests early and Modified Rodnan Skin Scores (MRSS) correlate with disease progression. Transcriptomics of SSc skin biopsies suggest the role of the *in vivo* microenvironment in maintaining the pathological myofibroblasts. Therefore, defining the structural changes in dermal collagen in SSc patients could inform our understanding of fibrosis pathogenesis. Here, we report a method for quantitative whole-slide image analysis of dermal collagen from SSc patients, and our findings of more aligned dermal collagen bundles in diffuse cutaneous SSc (dcSSc) patients. Using the bleomycin-induced mouse model of SSc, we identified a distinct high dermal collagen bundle alignment gene signature, characterized by a concerted upregulation in cell migration, adhesion, and guidance pathways, and downregulation of spindle, replication, and cytokinesis pathways. Furthermore, increased bundle alignment induced a cell migration gene signature in fibroblasts *in vitro*, and these cells demonstrated increased directed migration on aligned ECM fibers that is dependent on expression of *Arhgdib* (Rho GDP-dissociation inhibitor 2). Our results indicate that increased cell migration is a cellular response to the increased collagen bundle alignment featured in fibrotic skin. Moreover, many of the cell migration genes identified in our study are shared with human SSc skin and may be new targets for therapeutic intervention.

## Introduction

Systemic sclerosis (SSc) is a multifaceted disease encompassing vascular, autoimmune, and fibrotic components [1]. Distinct subsets of SSc have been described with varying severity; the two most well defined subsets termed limited cutaneous SSc (lcSSc) and diffuse cutaneous SSc (dcSSc) [2, 3]. In dcSSc, skin fibrosis can progress rapidly after onset of disease. The severity of skin disease, as measured by the Modified Rodnan Skin Score (MRSS), a clinical palpation method, has been shown to correlate well with fibrosis of internal organs and worse patient outcomes [4–7]. Interestingly, increased collagen accumulation and a morphological change to the dermal collagen organization has been reported in forearm skin biopsies from SSc patients. This change is characterized by a predominance of highly aligned collagen bundles, and a loss of the normal basket-weave collagen organization that is characteristic of the healthy dermis [8, 9]. Such observations of aligned collagen bundle organization have also been well documented in keloid scars [10], burn wounds [11], as well as in cases of physiological skin aging [12], and may be suggestive of a common underlying mechanism of tissue remodeling after injury and/or fibrosis. However, to the best of our knowledge, there has not been a robust and quantitative characterization of these structural changes in SSc skin, and therefore the evaluation of dermal collagen bundle alignment in relation to skin disease in SSc merits further investigation.

Transcriptomic profiling of SSc skin biopsies has revealed sets of pro-fibrotic genes strongly enriched in diseased as compared to normal biopsies [13–18]. However, transcriptomic analysis of explanted, *ex vivo* cultured SSc skin fibroblasts showed far fewer enriched genes as compared to normal fibroblasts [18]. These and other studies suggested the importance of the *in vivo* microenvironment in maintaining and supporting the pathological profile of SSc myofibroblasts. We posit that the well-organized ECM ultrastructure within the *in vivo* microenvironment could be important in maintaining the myofibroblasts phenotypes in SSc. Consistent with this idea, recent reports have highlighted the importance of the major fibroblastic collagen receptor α11β1 in the appearance of myofibroblasts during wound healing responses [19].

We addressed this hypothesis through a novel approach, combining the development and application of a method for quantitative image analysis of dermal collagen ultrastructure with genome-wide transcriptomic analysis. Our results indicate that collagen bundle alignment is a feature of dcSSc skin and is associated with a cell migration gene signature. Furthermore, we show that cell migration pathways are induced in primary human dermal fibroblasts cultured on aligned ECM fibers *in vitro*, and that these cells demonstrate increased directed migration on aligned ECM fibers that is dependent on expression of *Arhgdib* (Rho GDP-dissociation inhibitor 2).

## Materials and methods

### SSc skin samples

SSc forearm biopsy sections (3mm diameter punch biopsy) were obtained from the dermatopathology core of the National Scleroderma Core Center (Boston University). Samples analyzed included 6 healthy volunteers (HV), 5 limited cutaneous SSc (lcSSc), and 15 diffuse cutaneous SSc (dcSSc). Patients ages ranged from 25 to 71 years old, with a mean of 53.8 years. For a summary table of patient demographics, see (**Table A of** S1 File).

### Cell culture

Human dermal fibroblasts were obtained from ATCC (Manassas, VA). Cells were grown in Dulbecco’s modified eagle medium (DMEM) supplemented with 10% FBS and 1% Antibiotic-Antimycotic (ThermoFisher, Waltham MA). For *in vitro* assays on aligned versus random ECM fibers, cells were seeded at 500,000 cells/well into 60mm dishes with electrospun PCL nanofibers (Nanofiber Solutions Inc, Columbus OH) with adsorbed purified type I collagen (10ug/mL) overnight at 4°C, prior to cell seeding. Cells were cultured for 48 hours in a 37°C incubator, followed by total RNA extraction and whole genome transcriptomic analysis by RNASeq.

### Bleomycin-induced model of skin fibrosis in mice

All animal studies were conducted in strict accordance with the recommendations in the Guide for the Care and Use of Laboratory Animals of the National Institutes of Health, and in accordance with Cambridge (MA, USA) laws. The protocol was approved by the Institutional Animal Care and Use Committee of Biogen (protocol number: 516). All procedures were performed under isoflurane anesthesia, and all efforts were made to minimize suffering. The bleomycin model was established as described previously [20, 21], with daily subcutaneous injections of bleomycin (100uL at 1U/mL) into the back skin of 8 week old C57Bl/6 female mice, (N = 50 mice) (Jackson Labs, Bar Harbor, ME). At experimental endpoints, mice were euthanized with carbon dioxide, and back skin was harvested, with preservation of the anterior to posterior tissue orientation during tissue harvest. The biopsy was cut in half, with one half used for histopathology analysis, and the other half for RNASeq analysis.

### Image acquisition and analysis of dermal features

Picosirius red (PSR) staining was used for analysis of dermal collagen bundle alignment, according to the schematic shown in (**Figure A in** S1 File). Whole slide imaging was performed using both brightfield and polarized light microscopy at 40X magnification, using an Olympus VS120 scanner (Olympus Corp, Tokyo, Japan). Slides were digitized and individual collagen bundles identified using VisioPharm morphometry software (Ver 5.0.2, Copenhagen, Denmark). Analysis of dermis thickness (defined as area divided by contour length), total dermal collagen (measured as fraction of dermis with PSR staining), and bundle alignment was performed by scripting in Python and R.

### RNASeq, data analysis, and gene signature correlations

#### RNASeq procedure for mouse skin

Total RNA was isolated from skin biopsies, using GenElute mammalian total RNA kit (Sigma, St Louis, MO). Library preparation was done using TruSeq Stranded mRNA sample preparation kit (Illumina, San Diego, CA). Sequencing of 50bp paired-end reads was performed using an illumina HiSeq-2500, with an average coverage of 25 million reads/sample. Paired-end reads were aligned to the mm10 mouse reference genome using the STAR aligner, version 2.4.0; approximately 83% of all reads aligned uniquely to the genome. RNASeq analysis was performed with Bioconductor, using DESeq2 (Ver. 1.12.3). Criterion for assessing differentially expressed genes (DEG) was determined as follows: normalized counts > 3, |fold change| > 1.5, and adjusted p-value < 0.05, Benjamin Hochberg (BH) correction. A total of 4376 unique DEGs were identified following this criterion. Normalized counts of each expressed gene were correlated to each histopathological metric of skin fibrosis. Genes that met a threshold for correlation (r^2^ > 0.7, 0.4, 0.4 for dermis thickness, total collagen, and collagen bundle alignment, respectively) were then intersected with DEGs to identify gene signatures that captured each respective aspect of skin fibrosis. Pathway analysis was performed using GOStats package (Ver. 2.38.0) in Bioconductor. Visualization of interactions amongst enriched genes in the collagen bundle alignment signature was performed using STRINGdb. Analysis results of all genes can be found in (S1 Table). This dataset has been deposited into NCBI’s Gene Expression Omnibus and is accessible through GEO accession number GSE100212.

#### RNASeq procedure for human dermal fibroblasts

Dermal fibroblasts were cultured for 48 hours, on collagen-adsorbed aligned nanofibers vs. randomly oriented nanofibers coated with type I collagen. Total RNA isolation and library preparation was similar to process developed for skin tissue. Samples were sequenced to an average coverage of 29 million reads/sample. Paired-end reads were aligned to the hg19 human reference genome using STAR aligner, v2.4.0; approximately 90% of all reads aligned uniquely to the reference genome. RNASeq analysis was performed with Bioconductor, using DESeq2 (Ver. 1.12.3). DEG criterion: normalized counts >4, |fold change| > 1.5, and adjusted p-value < 0.05, Benjamin Hochberg (BH) correction. A total of 78 unique DEGs were identified following this criterion. Pathway analysis was performed using GOStats package (Ver. 2.38.0) in Bioconductor. Analysis results of all genes can be found in (S2 Table). This dataset has been depositied into NCBI’s Gene Expression Omnibus and is accessible through GEO accession number GSE99999 (https://www.ncbi.nlm.nih.gov/geo/query/acc.cgi?acc=GSExxx).

#### Cell migration experiments on *in vitro* nanofibers

Human dermal fibroblasts were seeded at a density of 3,500 cells per well in a 96 well plate coated with either aligned or randomly-oriented nanofibers, and adsorbed collagen type I. After 24 hours of culture, cells were loaded with CellTracker Red CMTPX dye, and imaged at 8 minute intervals on a PerkinElmer Phenix microplate for 4.5 hours. Cells were identified and tracked over time using Harmony software (V 4.0), and mean cell displacement and total accumulated distances were computed for all wells and experimental groups. In experiments with siRNA inhibition of Arhgdib, cells were transfected with 3 independent silencer select siRNA molecules for Arhgdib, or with negative control siRNA following manufacturer protocols (Thermo-Fisher, Waltham, MA). Next day after transfection, cells were harvested and seeded in 96 well plates, and cell migration experiments were performed as described above.

## Results

### Increased collagen bundle alignment is a feature of skin fibrosis in dcSSc

We aimed to develop a robust and unbiased method to quantitatively assess dermal collagen architecture in human SSc skin. As a direct method to assess collagen bundle alignment, we developed image analysis algorithms to identify all collagen bundles within the dermis, and computed the angular difference between each collagen bundle and the epidermis (**Figure A in** S1 File). Using this method, normal unaffected skin is characterized by two populations of collagen bundles at distinct angular directions, indicative of a basket-weave architecture. In contrast, skin that is predominantly composed of aligned collagen bundles in the dermis shows only a single population for angle distribution.

**Fig 1:**
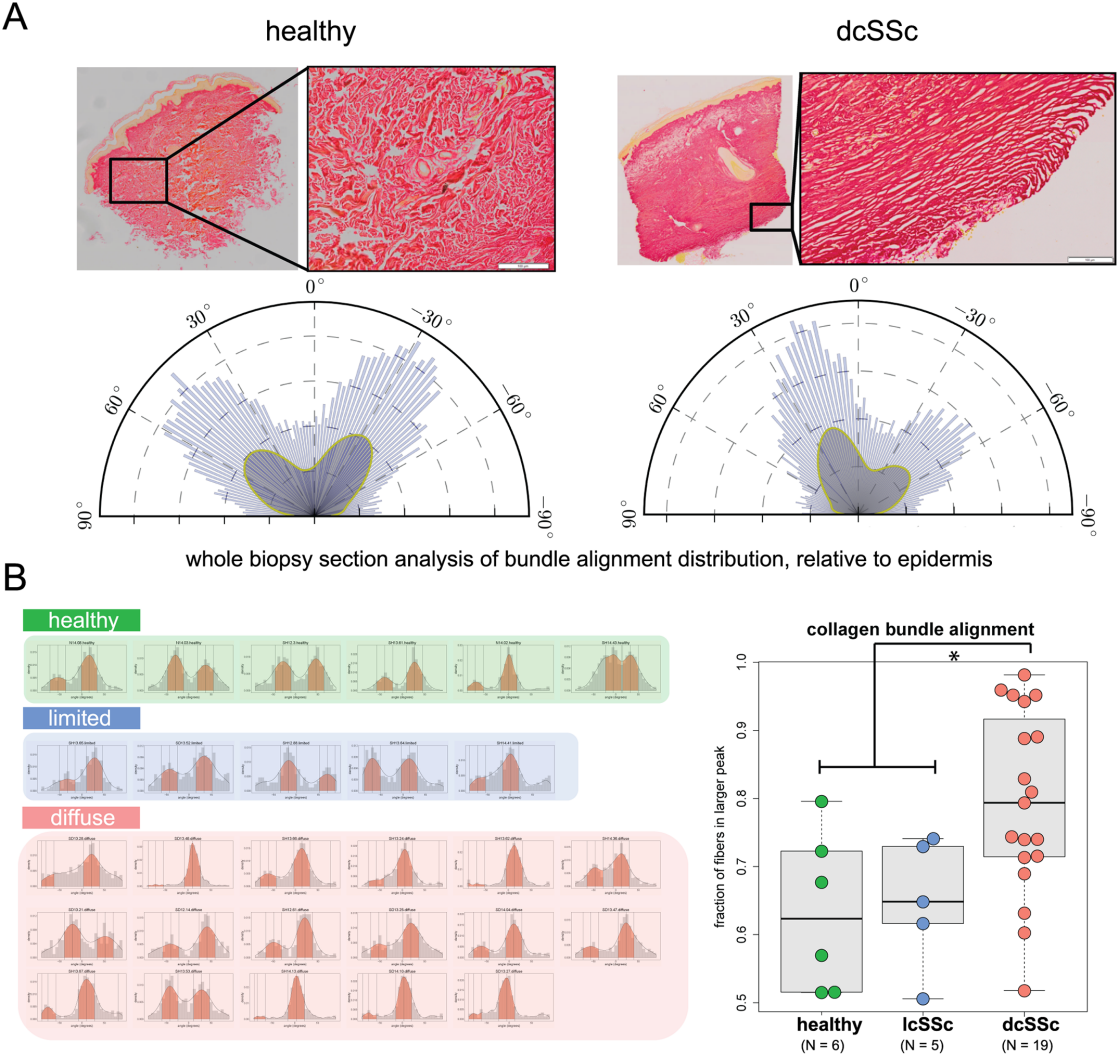
Collagen bundle alignment is a histopathological feature of dermal fibrosis in diffuse cutaneous SSc (dcSSc). **(A)** Representative image of a forearm skin biopsy from (left) healthy and (right) dcSSc subjects (scale bar = 100μm). Individual dermal collagen bundles were identified in PSR stained tissue sections, and the angle of alignment for each bundle across the whole biopsy section were computed relative the epidermis using automated algorithms in VisioPharm software. Angle distributions shown relative to the epidermis (0 degrees) and **(B)** as a histogram for each of 6 healthy, 5 lcSSc and 17 dcSSc subjects (y-axis label: density, x-axis label: angle relative to epidermis). Analysis of the healthy samples shows a characteristic bimodal distribution, whereas dcSSc samples show a distribution that is unimodal or skewed to one peak. Quantification of collagen bundle alignment as the relative proportion of bundles in each peak (fraction of fibers within the greater of the two peaks) are shown as boxplots for healthy, lcSSc and dcSSc. (Boxplot represent first quartile, median, and third quartile. Whiskers show min to max. *, p < 0.05 by ANOVA, error bars are SEM).

We applied these methods to the study of forearm skin biopsies from SSc patients (see **Table A in** S1 File) for a summary of the patient demographics). We found that patients with dcSSc have significantly higher alignment of dermal collagen bundles as compared to those with lcSSc and healthy volunteers (HV), (median alignment = 0.79 ± 0.031 SEM) compared to healthy controls (median alignment = 0.62 ± 0.048 SEM) (Fig 1). Dermal collagen from healthy volunteers generally showed the expected basket-weave architecture, characterized by a bimodal distribution in bundle alignment angles (Fig 1A). In contrast, dcSSc patients showed more variability in bundle alignment, with a subset of patients presenting with highly aligned dermal collagen bundles, and other patients presenting with more normal bundle alignment.

Analysis of biopsies from patients with lcSSc showed less pathological alignment, and a preservation of the characteristic basket-weave pattern of healthy skin. The dcSSc patients also have significantly increased total dermal collagen as compared to samples from lcSSc patients and healthy volunteers (**Figure B in** S1 File), measured as the fractional area of the dermis with positive collagen staining. Interestingly, there is a moderate correlation (R^2^ = 0.48) between collagen bundle alignment and total dermal collagen, suggesting that bundle alignment is associated with accumulation and deposition of dermal collagen. We did not observe any significant correlations of collagen bundle alignment with age or total skin score (MRSS) (**Figure B in** S1 File), indicating that the differences in bundle alignment is a distinct feature independent of these factors. In a subset of dcSSc patients where serial FFPE sections were available, we performed aSMA staining to identify myofibroblasts (**Figure C in** S1 File). Prominent myofibroblast accumulation was observed in 2 of 7 samples analyzed, with spindle-shaped aSMA+ stained cells primarily localized to the deep dermis, in close proximity to the highly aligned collagen fiber bundles. Incidentally, the 2 patients that showed the prominent myofibrblast staining where also the patients with the highest collagen bundle alignment by our measures (alignment scores of 0.98 and 0.95, respectively).

### Mouse models of SSc recapitulated aspects of human SSc by collagen architecture analysis

To gain a better mechanistic understanding of the pathophysiological significance of dermal collagen bundle alignment, we aimed to assess dermal collagen architecture in a mouse model of SSc, wherein we could simultaneously collect tissue for image analysis as well as whole-genome transcriptomic analysis. We used the well characterized bleomycin-induced dermal fibrosis model of SSc in which dermal thickness and total dermal collagen (fractional area of the dermis with positive collagen staining) are increased at 2–4 week of bleomycin treatment [20, 22]. Additionally, we included experimental groups in which bleomycin treatment was stopped at 4 weeks. These animals were then allowed to recover for an additional 6 or 10 weeks, to assess if aspects of the histopathology induced by bleomycin treatment could be normalized.

**Fig 2:**
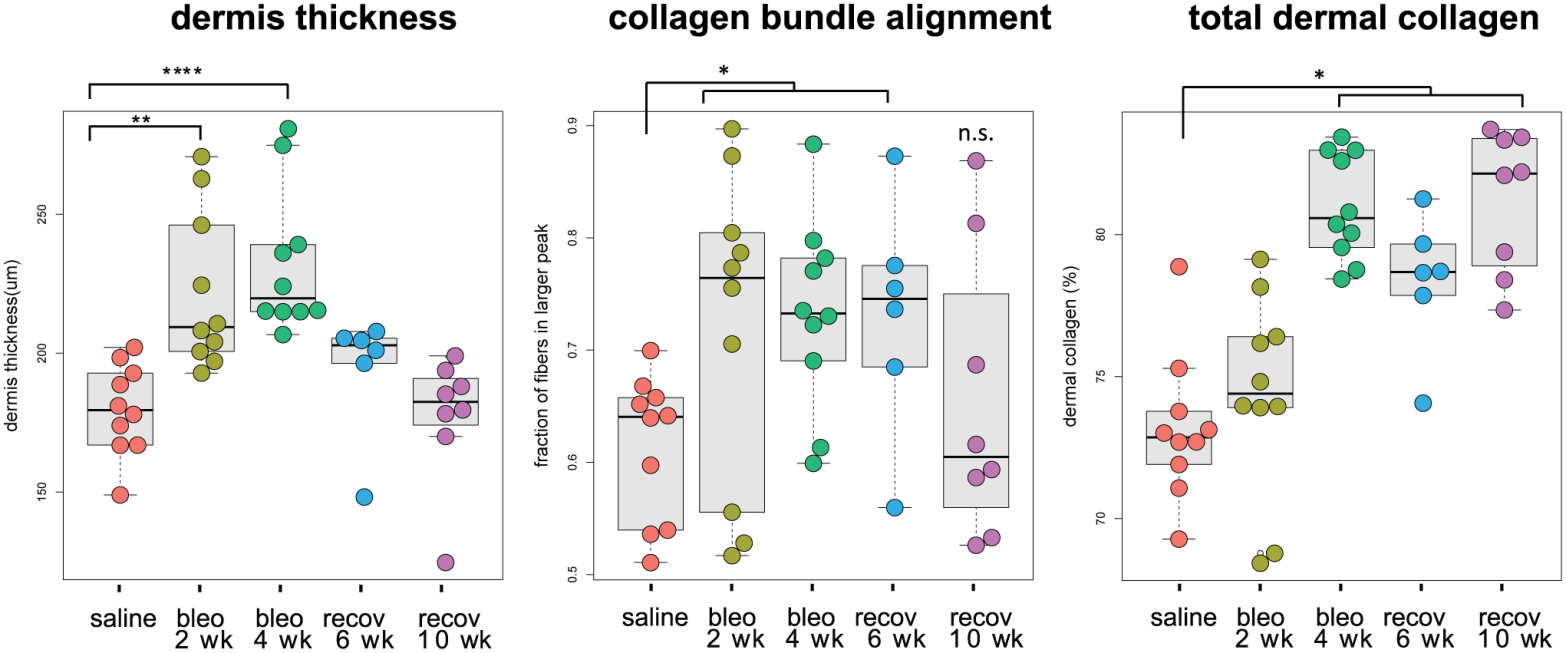
Mouse bleomycin-induced model of SSc recapitulates the collagen bundle alignment feature of human SSc dermal architecture. Quantification of dermal thickness (left), collagen bundle alignment (center), and total collagen within the dermis (right) for cohorts (n = 6-10/group) injected daily s.c. with saline, or bleomycin for 2 weeks, 4 weeks, or for 4 weeks and then untreated for an additional 6 or 10 weeks (recovery 6 wk and 10 wk). All analysis was performed on whole biopsy sections (shown in **Figure D of** S1 File), using automated algorithms in VisioPharm software. (* p < 0.05, ** p < 0.01, *** p < 0.001 by ANOVA, error bars are SEM).

Dermal thickness was the earliest histopathological change, and was significantly increased by 2 weeks of bleomycin treatment, and peaked at 4 weeks of treatment. Dermal thickness completely normalized to control levels at both the 6 and 10-week recovery time points (Fig 2). Total dermal collagen content (assessed as fraction of dermis with PSR staining), was also significantly increased at 4 weeks of bleomycin treatment, but did not normalize with recovery. Importantly, we found increased dermal collagen bundle alignment with bleomycin treatment, a novel observation that has not been previously reported, demonstrating that the mouse model recapitulates this distinct feature of human dcSSc skin (Fig 2). Increased collagen bundle alignment with bleomycin treatment was evident at 2 weeks, peaked at 4 weeks, and normalized to baseline levels with recovery. Together, our analysis revealed that total collagen deposition is preceded by an increase in both dermis thickness and collagen bundle alignment, and suggests that these earlier changes to the dermal collagen architecture may play a role in supporting or enhancing the later collagen deposition.

The different temporal kinetics of each histopathological change suggested that dermis thickness, total dermal collagen, and collagen bundle alignment constitute diverse aspects underlying fibrosis progression and recovery. Consistent with this, we found no significant correlations amongst the three different features (**Figure E of** S1 File), suggesting that measurements of these features complement each another in describing the complex structural changes to the skin with ongoing fibrosis.

### Transcriptomic analysis reveals distinct core gene expression signatures associated with dermal thickness, total collagen content and collagen bundle alignment

We performed genome-wide transcriptomic analysis using the other half of the same biopsy samples we analyzed for histology. This enabled us to correlate the expression of differentially expressed genes (DEG) with the different features of the dermal architecture, and thereby inform the molecular profiles underlying these pathological changes.

**Fig 3:**
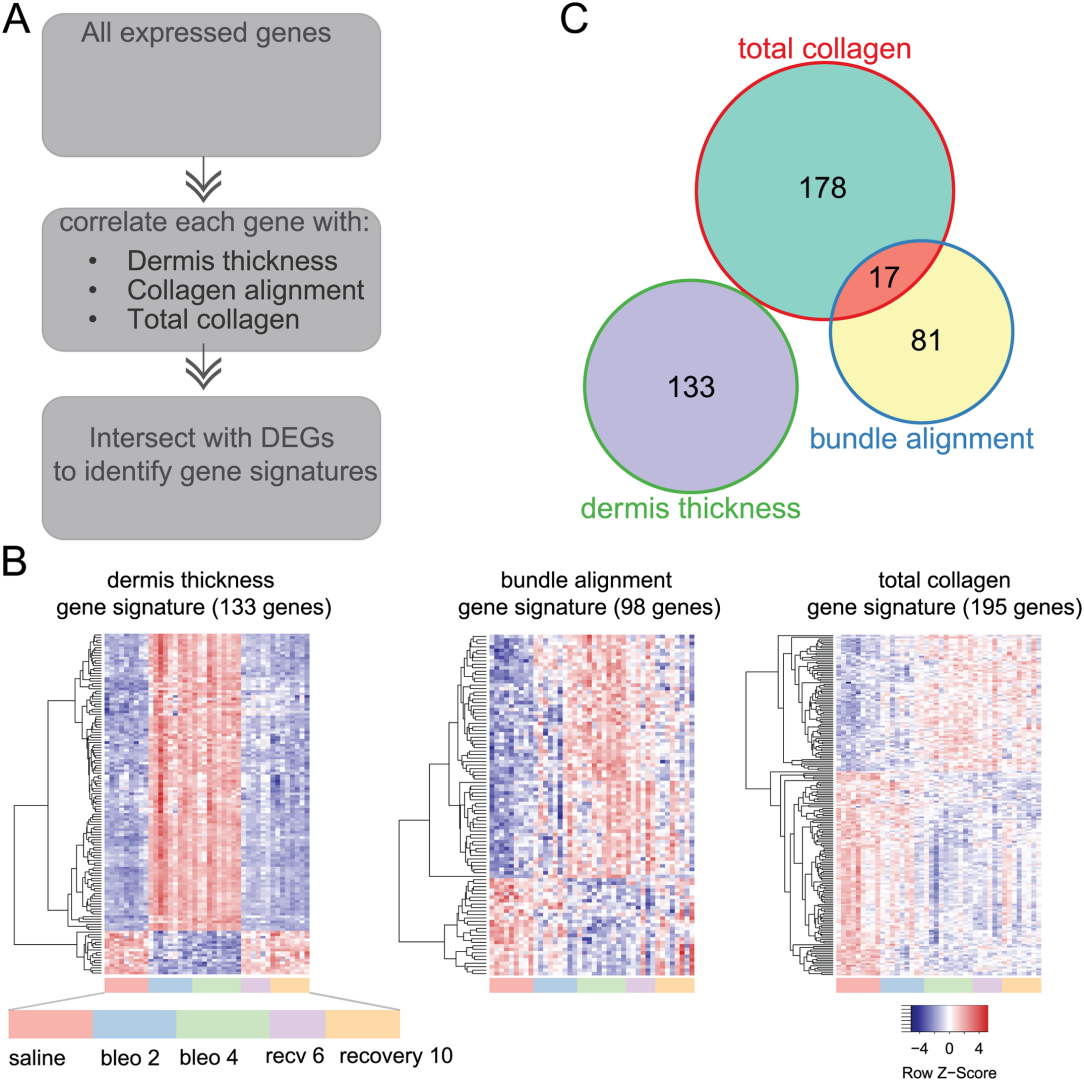
Histopathology and transcriptomic analysis reveals distinct core gene expression signatures of dermal thickness, collagen bundle alignment and total collagen. **(A)** Workflow for identifying gene signatures associated with collagen architectural changes with bleomycin-induced fibrosis. Samples for transcriptomic analysis were obtained from the same study as those for histopathological analysis as described in Fig 2. Across all samples analyzed, expression level of each gene was correlated to histopathological measurements of dermis thickness, collagen bundle alignment, and total collagen. Gene with sufficient correlations were then intersected with pooled differentially expressed genes (DEGs), ie. genes that fulfilled differential expression criteria in any of the comparisons between experimental groups vs. saline control (see Methods), to identify gene signatures. **(B)** Heatmaps showing the expression level of distinct gene signatures identified for each of the dermal architectural features. These signatures could appreciably differentiate amongst treatment groups. Dermal thickness signature shows clear differentiation between 2 and 4 week bleomcyin-treated groups (bleo 2 and bleo 4) from saline and recovery groups (recv 6 and recv 10). Bundle alignment and total collagen signatures separate saline groups from all other groups. **(C)** Pathway analysis by gene ontology (GO) enrichment was performed with GOstats package in Bioconductor. GO term enrichment showed minimal overlap among the three distinct gene signatures. Dermis thickness gene signature was associated with innate immune and interferon signaling; collagen bundle alignment signature enriched for cell membrane components, and total collagen gene signature enriched for mitosis and cell cycle processes.

The expression levels of each expressed gene were first correlated with the levels of dermal thickness, collagen bundle alignment, and total collagen content, across all samples analyzed (Fig 3A). These correlated genes were then intersected with DEG, defined as genes that fulfilled differential expression criteria in any of the comparisons between experimental groups vs. saline control. This analysis identified gene signatures associated with each aspect of fibrotic skin (dermal thickness, collagen bundle alignment, and total dermal collagen). Using this approach, we identified a gene signature associated with dermis thickness that robustly differentiated the saline, bleomycin-treated and recovery groups. We also identified distinct gene signatures associated with collagen bundle alignment and total dermal collagen, and discovered that these signatures were more similar to each other as compared to the gene signature associated with dermis thickness (Fig 3B), suggesting likely interactions between the molecular mechanisms underlying bundle alignment and collagen deposition. Both the total collagen and collagen bundle alignment gene signatures appreciably separate saline from bleomycin and recovery samples, but not bleomycin-treated samples from recovery samples.

Pathway analysis of signature genes associated with dermal thickness showed significant enrichment of innate immune cell signaling pathways and pathways associated with anti-viral responses consistent with prior literature associating an inflammatory signature with the bleomycin model[14, 16]. This supports an interpretation that increased dermis thickness early after bleomycin treatment is indicative of inflammatory responses to tissue injury, particularly interferon (IFN) signaling. Genes uniquely associated with total dermal collagen enriched for a large number of pathways, with most significant enrichment in pathways associated with cell cycle processes, nuclear division, and mitosis (data not shown). In contrast, examination of the 81 genes uniquely associated with collagen bundle alignment identified a core set of 7 genes with shared protein-protein interactions. These genes are known to play import roles in cell adhesion, cell migration, and guidance of neural and vascular processes (**Figure F of** S1 File), and their expression was significantly upregulated in concert following bleomycin challenge. Notably, 5 of the 7 genes were also featured in published human SSc datasets (*Emcn*, *Esam*, *Gfra3*, *Robo4*, *and Sparcl1*). Furthermore, the expression levels of these genes were all positively correlated with bundle alignment (**Figure G of** S1 File**)**. Together, these data suggested that this set of genes may play a role in directing the cellular response to fibrosis-induced collagen bundle alignment.

### Alignment of ECM fiber bundles alters gene expression signatures of human dermal fibroblasts and enhances cell migration phenotype

**Fig 4:**
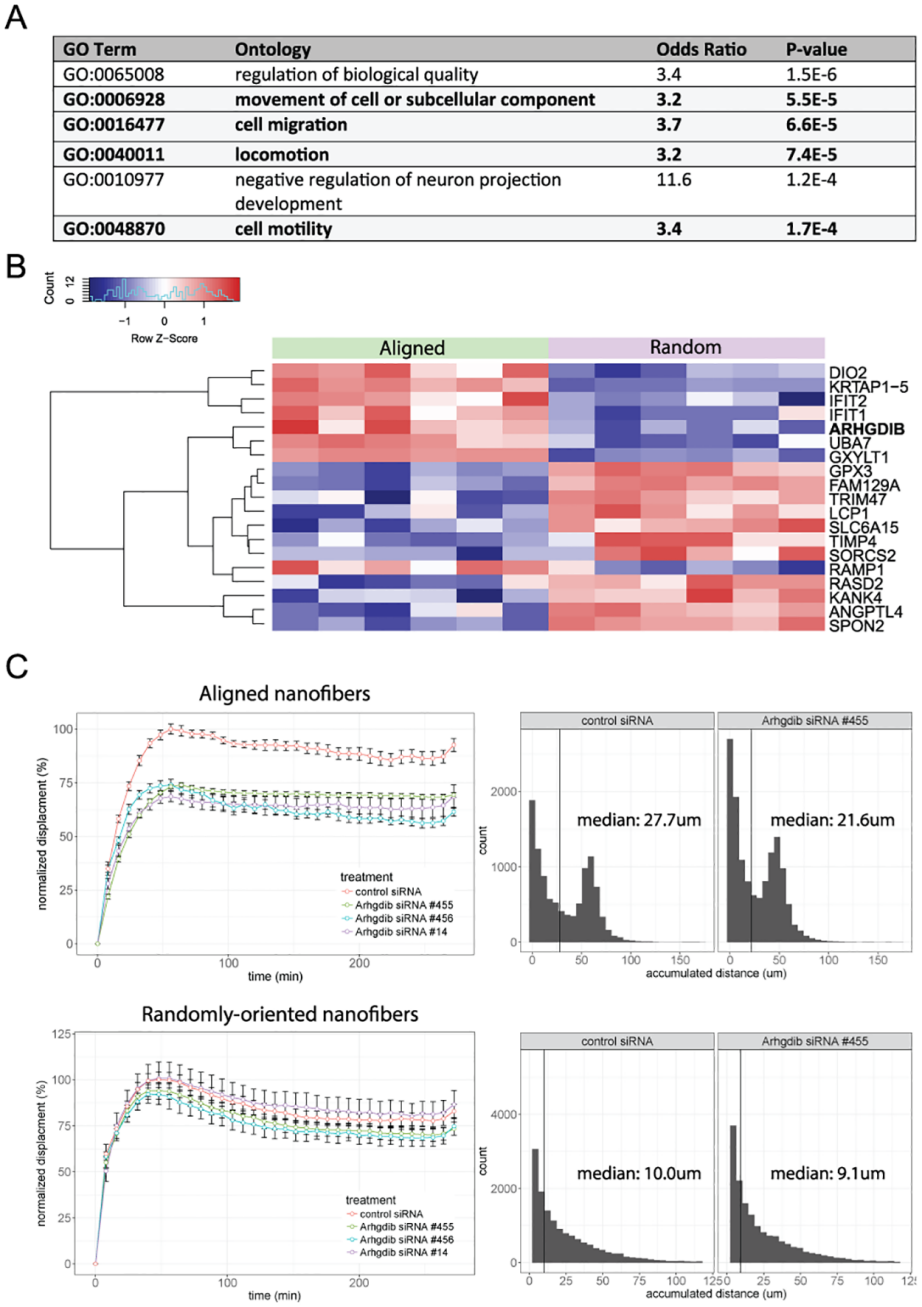
Primary human dermal fibroblasts respond to aligned ECM fibers by alterations in cell migration signaling. Dermal fibroblasts were cultured for 48 hours on aligned vs. randomly oriented nanofibers coated with type I collagen. RNAseq was performed on harvested. A total of 78 genes exhibited differential expression between the two culture conditions. **(A)** Gene ontology (GO) enrichment of DEGs. GO over-representation was performed with GOstats package in Bioconductor. Top enriched ontologies shown, ordered by p-value. **(B)** Heatmap representation using normalized FPKM values of the 19 DEGs that were also featured in published human SSc datasets. Each column represents an independent biological replicate. **(C)**. Assay of fibroblast migration by time lapse imaging. Cells were transfected with silencer select siRNA (ThermoFisher, Waltham, MA) against *Arhgdib* using 3 independent siRNAs, or a negative control siRNA. Mean cell displacement (left panels) and accumulated migration distances (right panels) on aligned-nanofibers (top row) or randomly-oriented nanofibers (bottom row). Mean cell displacement is normalized to control siRNA transfected samples. Mean cell displacement was reduced by approximately 25% upon Arhgdib inhibition on aligned fiber culture conditions (p < 7.0e-11). Results are show for 1 of 2 representative experiments. No significant effect was seen in cells cultured on randomly-oriented fibers.

To directly determine whether collagen bundle alignment can affect the phenotypes of dermal fibroblasts, we used an *in vitro* model for 3D cell culture in systems of either randomly oriented or aligned nanofibers with adsorbed type I collagen. Expression profiling of human dermal fibroblasts from these culture conditions identified enrichment of cell migration-related pathways associated with the aligned fiber condition (Fig 4A). Although the specific genes identified here differ from those found in the bleomycin model bundle alignment signature, both are cell migration-related. Additionally, we performed time lapse imaging using the same *in vitro* culture systems, and observed that fibroblasts cultured on aligned nanofibers with adsorbed collagen showed an enhanced migratory phenotype compared to fibroblasts on randomly-oriented collagen-adsorbed nanofibers (**Figure H of** S1 File), with increased migratory distance and directionality.

Of the 78 differentially expressed genes identified from our *in vitro* model, 19 of these were also expressed in at least one of three published SSc skin datasets (Fig 4B, Table 1, **Table B of** S1 File). *Arhgdib* (Rho GDP-dissociation inhibitor 2) was one of the most upregulated genes following fibroblast culture on aligned fiber substrates and significantly upregulated in 2 separate published SSc skin datasets, as well as in our bleomycin model. Therefore, we evaluated the potential role of *Arhgdib* in regulating fibroblast migration on aligned vs. randomly-oriented ECM fibers. Knockdown of *Arhgdib* by RNA silencing significantly reduced directed cell migration under aligned fiber culture conditions, as shown by reduced mean cell displacement (Fig 4C, **Figure I of** S1 File). In contrast, *Arhgdib* inhibition had no effects on mean cell displacement when cells were cultured on randomly-oriented fiber substrates. These data indicate a role for *Arhgdib* in directed cell migration on aligned ECM fibers.

**Table 1:** DEGs in primary human fibroblasts cultured on aligned vs. randomly-oriented fibers that are shared with human SSc datasets.

Symbol	Name	Functions	SSc dataset*			
			1	2	3	4
DIO2	deiodinase, iodothyronine, type II	Thyroid hormone action, thyroxine to T3 conversion	✓	✓		✓
KRTAP1-5	keratin associated protein 1–5	Structural protein in hair shaft	✓			✓
IFIT2	Interferon-Induced Protein With Tetratricopeptide Repeats 2	Interferon signaling, Anti-viral response, RNA binding			✓	✓
IFIT1	Interferon-Induced Protein With Tetratricopeptide Repeats 1	Interferon signaling, Anti-viral response, RNA binding			✓	✓
ARHGDIB	Rho GDP dissociation inhibitor (GDI) beta	Cell migration, GTPase activity	✓	✓		✓
UBA7	Ubiquitin-Like Modifier Activating Enzyme 7	E1 ubiquitin-activating enzyme, interferon signaling, antigen processing			✓	✓
GXYLT1	Glucoside Xylosyltransferase 1	Metabolism, Heparan sulfate/heparin (HS-GAG) metabolism			✓	
GPX3	Glutathione Peroxidase 3	Glutathione metabolism, Cellular Senescence	✓			✓
FAM129A	Family With Sequence Similarity 129, Member A	response to endoplasmic reticulum stress			✓	
TRIM47	Tripartite Motif Containing 47	metal ion binding	✓			
LCP1	Lymphocyte Cytosolic Protein 1	T cell activation involved in immune response, Cell migration			✓	✓
SLC6A15	Solute Carrier Family 6 (Neutral Amino Acid Transporter), Member 15	ion transport, neurotransmitter transpor			✓	
TIMP4	TIMP metallopeptidase inhibitor 4	Extracellular matrix	✓			✓
SORCS2	sortilin related VPS10 domain containing receptor 2	Unknown, biomarker for amyotrophic lateral sclerosis	✓			
RAMP1	Receptor (G protein-coupled) activity modifying protein 1	Calcitonin receptor binding, T-cell activation	✓			
RASD2	RASD family member 2	Locomotory behavior, GTPase activity	✓			✓
KANK4	KN motif and ankyrin repeat domains 4	Cell motility	✓			
ANGPTL4	angiopoietin like 4	Lipid metabolism, angiogenesis, anti-apoptosis	✓			✓
SPON2	spondin 2	Cell migration, innate immunity	✓			✓

DEG were identified in primary human fibroblasts as described in Fig 4 and intersected with those from publically available human SSc datasets and our murine bleomycin study herein

*datasets referenced: 1. [13], 2. [16] 3. [18] 4. Mouse model of bleomycin-induced SSc herein

## Discussion

It has been posited that the well-organized ECM ultrastructure within the *in vivo* microenvironment could be important in maintaining the pathological myofibroblast phenotype in SSc. We addressed this hypothesis through a novel approach, using our newly developed quantitative method of image analysis of skin collagen ultrastructure in conjunction with genome-wide transcriptomic analysis. We quantified collagen bundle alignment as a feature of dcSSc in forearm skin biopsies and showed it to be recapitulated in mouse models of SSc. Furthermore, we also identified gene expression pathways correlated with this feature, suggesting that the guidance cues from aligned collagen bundles enhance the fibrogenic potential of dermal fibroblasts, via increased signaling through cell migration, adhesion, and guidance pathways, and we supported this thesis by directly validating that fiber alignment enhances the directed migration of human dermal fibroblasts in engineered 3D culture systems with aligned nanofibers vs randomly oriented nanofibers. Importantly, we also demonstrate a fiber alignment-induced cell migration gene signature, including DEGs also upregulated in human SSc skin, and a role for *Arhgdib* in regulating cell migration on aligned but not randomly-oriented ECM fibers. Together, these results suggest that collagen bundle alignment in dcSSc patients may be an extracellular cue in fibrotic ECMs to which fibroblasts/myofibroblasts respond by altering their cell migration phenotype.

We quantitatively established that dermal collagen bundle alignment is increased in dcSSc skin and that this feature is recapitulated in the bleomycin model of SSc in mice. These observations are not limited to the bleomycin model, as we also observed increased collagen bundle alignment in the sclerodermatous graft-vs-host disease model of SSc (scl-GVHD), suggesting that changes to collagen bundle alignment is likely a feature of dermal fibrosis and not unique to one model (**Figure J of** S1 File).

Our *in vivo* results suggested that increased collagen bundle alignment with fibrosis progression coordinates changes in gene expression to promote cell migration and inhibit cell division. This interpretation is also supported by literature on the effects of substrate alignment cues on cell migration and motility [23–26]. To directly validate the effects of ECM alignment on primary human fibroblast motility and additionally elucidate the gene expression changes induced by fiber alignment, we employed an *in vitro* system whereby these cells were cultured in a 3D culture system with either randomly-oriented or aligned nanofibers adsorbed with type I collagen. Although the simplicity of this system limits its physiological relevance, this system allowed us to isolate the effects of ECM fiber bundle alignment from the myriad of other complexities within the *in vivo* microenvironment. Using this system, we identified an enrichment of cell migration related pathways that was associated with aligned-fiber culture conditions. Notably, when we intersected the 78 DEG identified in the fibroblasts cultured on the aligned vs. randomly-oriented nanofibers with the DEG in human dcSSc vs healthy skin datasets, we found that 24% of the DEGs (19 of 78) are also upregulated in at least one of three published human SSc datasets [13, 16, 18] (Table 1). Notable among these is *Arhgdib* (Rho GDP Dissociation Inhibitor Beta), a gene that has reported roles in regulating cell migration in lung and pancreatic tumors [27–29], and is also differentially expressed in published human SSc datasets. Additionally, we observed from time-lapse imaging experiments that fibroblasts on aligned fiber substrates showed an enhanced migratory phenotype, manifested as increased mean cell displacement and increased total migration distance. Using siRNA knockdown of *Arhgdib* in migration experiments (Fig 4C), we showed that inhibition of this gene resulted in reduced mean cell displacement of cells on aligned but not randomly-oriented nanofibers, suggesting that *Arhgdib* may be one of the genes mediating the specific cell migration response to aligned ECM fibers in SSc.

We also identified a cell migration gene signature associated with bundle alignment in the mouse bleomycin model, with 5 of the 7 signature genes also featured in human SSc. Although we observed that cell migration signaling was similarly enriched in the bundle-alignment signature in our analysis of both the bleomycin model *in vivo* and the aligned cultured system *in vitro*, we did not identify the same set of genes from both experiments. We believe that this discrepancy may be attributed to the inherent differences between the two experimental systems (*in vivo* tissue vs. *in vitro* cell culture) as well as differences in species (mouse vs. human). Despite these differences, the association between collagen bundle alignment and cell migration genes suggests a broadly conserved cellular response to increased alignment of ECM fibers within fibrotic skin.

One important limitation of our current study is the small sample size of the SSc cohort analyzed. Another limitation is that we were not able to validate the genes of interest (identified by RNASeq) by immunostaining of human SSc skin samples due to limited SSc sample availability. Because SSc is a highly heterogeneous disease, future studies using larger samples sizes, and well defined clinical cohorts will be necessary to confirm these findings.

Based on our novel approach encompassing structural, transcriptomic and functional analyses, we identified genes and pathways that appeared to be dependent on collagen bundle alignment, that are also featured in human SSc skin datasets, therefore suggesting that responses to collagen bundle alignment may be an important factor in the fibrogenic process in SSc. We propose that ECM fiber bundle alignment acts as an extracellular cue directing fibroblast migration, and that dysregulation of the organized ECM ultrastructure may thereby be a driver in fibrotic diseases such as SSc. One possible mechanism is through induced cell polarization by extensive interactions with the highly aligned underlying substrate. Indeed, such a mechanism has been observed in vitro, whereby persistent, high velocity motility of tumor cells is observed when cells are cultured on unidirectional micropatterned stripes, but not in 2D culture [30]. We speculate that there is crosstalk whereby the migratory fibroblasts in turn play a role in shaping the ECM. Indeed, within the fibrotic regions in SSc skin, we and others [31] have also observed fibroblasts embedded within, and closely associated with densely packed collagen bundles—suggesting that a similar mechanism may also be relevant *in vivo*. Understanding these cell-ECM interactions may inform our understanding of the pathogenesis of SSc and may lead to new therapeutic targets for treatment of this intractable disease.

## Supporting information

S1 File Supporting information. Compiled supplementary figures and tables.
(DOCX)

S1 Table Differentially expressed genes from bleomycin mouse model. (CSV)

S2 Table Differentially expressed genes from human fibroblast culture. (CSV)
