# Supplementary material for: Increased dermal collagen bundle alignment in systemic sclerosis is associated with a cell migration signature and role of Arhgdib in directed fibroblast migration on aligned ECMs
Source: PLoS One. 2017 Jun 29;12(6):e0180751. doi: 10.1371/journal.pone.0180751 (PMC5491269; doi:10.1371/journal.pone.0180751)
Supplement: S1 File — Compiled supplementary figures and tables. (DOCX) [file pone.0180751.s001.docx]

**Supporting Information**

**Table A.** Human SSc skin biopsies: demographics and clinical parameters

|  | **Diffuse (n = 19)** | **Limited (n = 5)** | **Healthy (n = 6)** |
| --- | --- | --- | --- |
| **Age (mean ± SD years)** | 53.6 ± 10.3 | 61.8 ± 4.2 | 41.2 ± 16.1 |
| **Disease duration (mean ± SD months)** | 22.4 ± 29.4 | 51.5 ± 78.0 | N/A |
| **Gender (male, female), (% male)** | (7, 12) (0.35) | (0, 5) (0) | (5, 1) (0.83) |
| **Total body MRSS (mean ± SD)** | 24.1 ± 12.6 | 2.5 ± 1 | N/A |

**Table B.** DEGs in primary human fibroblasts cultured on aligned vs. randomly-oriented fibers that are shared with human SSc datasets. Fold change and adjusted p-values indicated, N = 6 for each group (aligned vs. random).

| **Symbol** | **Name** | **Functions** | **fold change** | **p-adj** |
| --- | --- | --- | --- | --- |
| DIO2 | deiodinase, iodothyronine, type II | Thyroid hormone action, thyroxine to T3 conversion | 1.507199 | 9.06E-09 |
| KRTAP1-5 | keratin associated protein 1-5 | Structural protein in hair shaft | 1.530911 | 2.41E-14 |
| IFIT2 | Interferon-Induced Protein With Tetratricopeptide Repeats 2 | Interferon signaling, Anti-viral response, RNA binding | 1.61875 | 4.98E-05 |
| IFIT1 | Interferon-Induced Protein With Tetratricopeptide Repeats 1 | Interferon signaling, Anti-viral response, RNA binding | 1.628676 | 7.27E-07 |
| ARHGDIB | Rho GDP dissociation inhibitor (GDI) beta | Cell migration, GTPase activity | 1.537046 | 2.50E-05 |
| UBA7 | Ubiquitin-Like Modifier Activating Enzyme 7 | E1 ubiquitin-activating enzyme, interferon signaling, antigen processing | 1.503776 | 1.15E-08 |
| GXYLT1 | Glucoside Xylosyltransferase 1 | Metabolism, Heparan sulfate/heparin (HS-GAG) metabolism | 1.600852 | 3.01E-24 |
| GPX3 | Glutathione Peroxidase 3 | Glutathione metabolism, Cellular Senescence | 0.551699 | 1.51E-06 |
| FAM129A | Family With Sequence Similarity 129, Member A | response to endoplasmic reticulum stress | 0.662971 | 1.59E-13 |
| TRIM47 | Tripartite Motif Containing 47 | metal ion binding | 0.646986 | 0.002053 |
| LCP1 | Lymphocyte Cytosolic Protein 1 | T cell activation involved in immune response, Cell migration | 0.601185 | 0.000847 |
| SLC6A15 | Solute Carrier Family 6 (Neutral Amino Acid Transporter), Member 15 | ion transport, neurotransmitter transpor | 0.656139 | 2.93E-05 |
| TIMP4 | TIMP metallopeptidase inhibitor 4 | Extracellular matrix | 0.604752 | 0.008017 |
| SORCS2 | sortilin related VPS10 domain containing receptor 2 | Unknown, biomarker for amyotrophic lateral sclerosis | 0.602808 | 0.016744 |
| RAMP1 | Receptor (G protein-coupled) activity modifying protein 1 | Calcitonin receptor binding, T-cell activation | 1.698746 | 0.044625 |
| RASD2 | RASD family member 2 | Locomotory behavior, GTPase activity | 0.571867 | 0.00014 |
| KANK4 | KN motif and ankyrin repeat domains 4 | Cell motility | 0.60567 | 0.000751 |
| ANGPTL4 | angiopoietin like 4 | Lipid metabolism, angiogenesis, anti-apoptosis | 0.610034 | 1.09E-05 |
| SPON2 | spondin 2 | Cell migration, innate immunity | 0.656147 | 9.11E-11 |

**Figure A.** Workflow for identification and quantification of dermal collagen bundle alignment
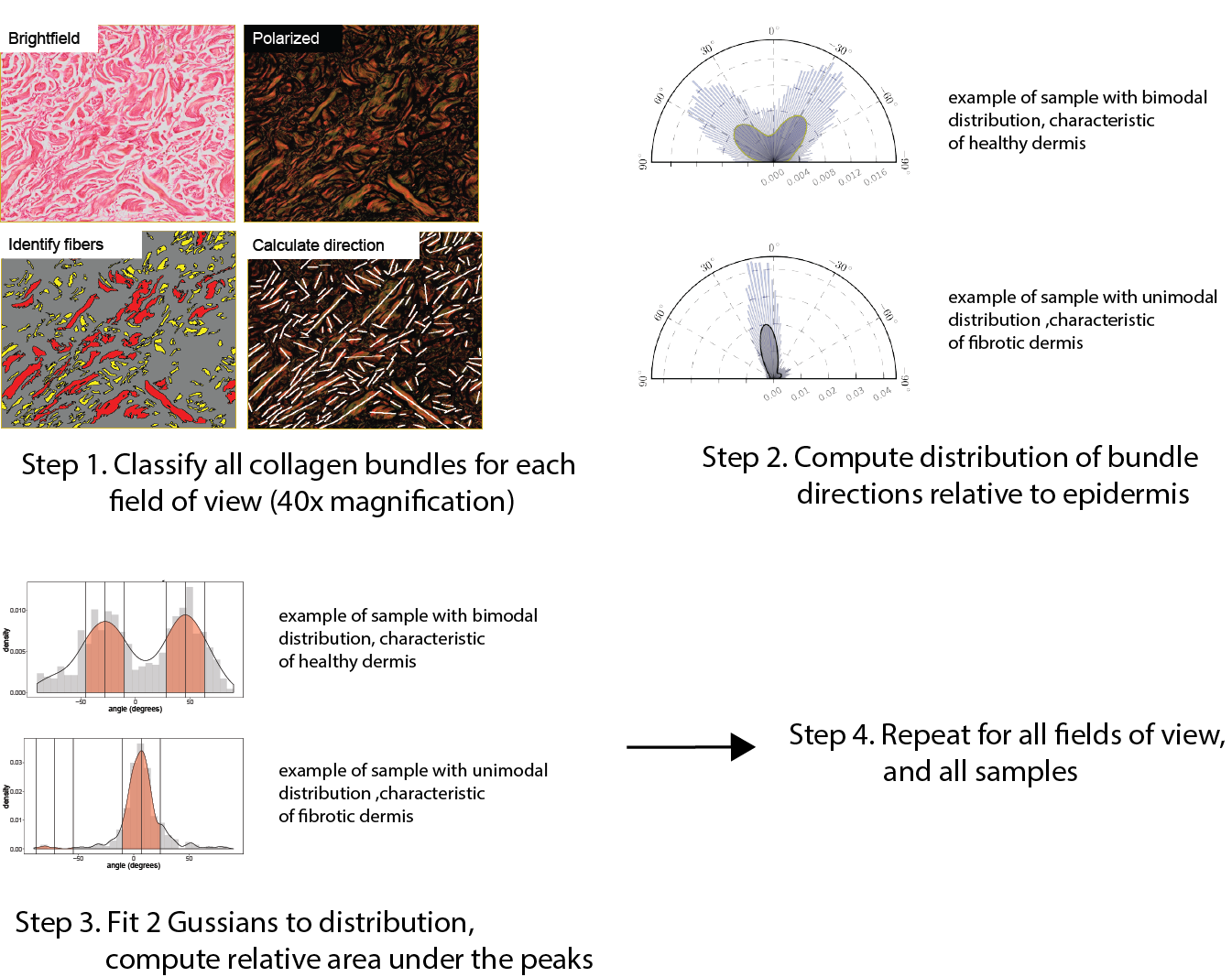


**Step 1)** Collagen bundles in forearm skin biopsy tissue sections were stained by picosirius red (PSR). Slides were digitally scanned, and whole slide imaging performed to visualize collagen bundles under circularly polarized light microscopy. **Step 2)** Angle of alignment of each collagen bundle was computed relative to the epidermis, and distributions of collagen bundle directions were analyzed for each sample. Healthy skin shows the characteristic bimodal distribution, whereas a single or skewed distribution was observed in dcSSc samples. **Step 3)** Quantification of collagen bundle alignment is defined as the relative proportion of fibers under each distribution. Healthy samples with a biomodal distribution will have a ratio close to 0.5, while skin with higher bundle alignment will have a ratio skewed towards 1.0 **Step 4)** Analysis described in steps 1-3 was repeated for all samples.

**Figure B.** Dermal collagen bundle alignment reflects a distinct aspect of fibrotic tissue that is associated with total collagen, but independent of Modified Rodnan Skin Score (MRSS) and age


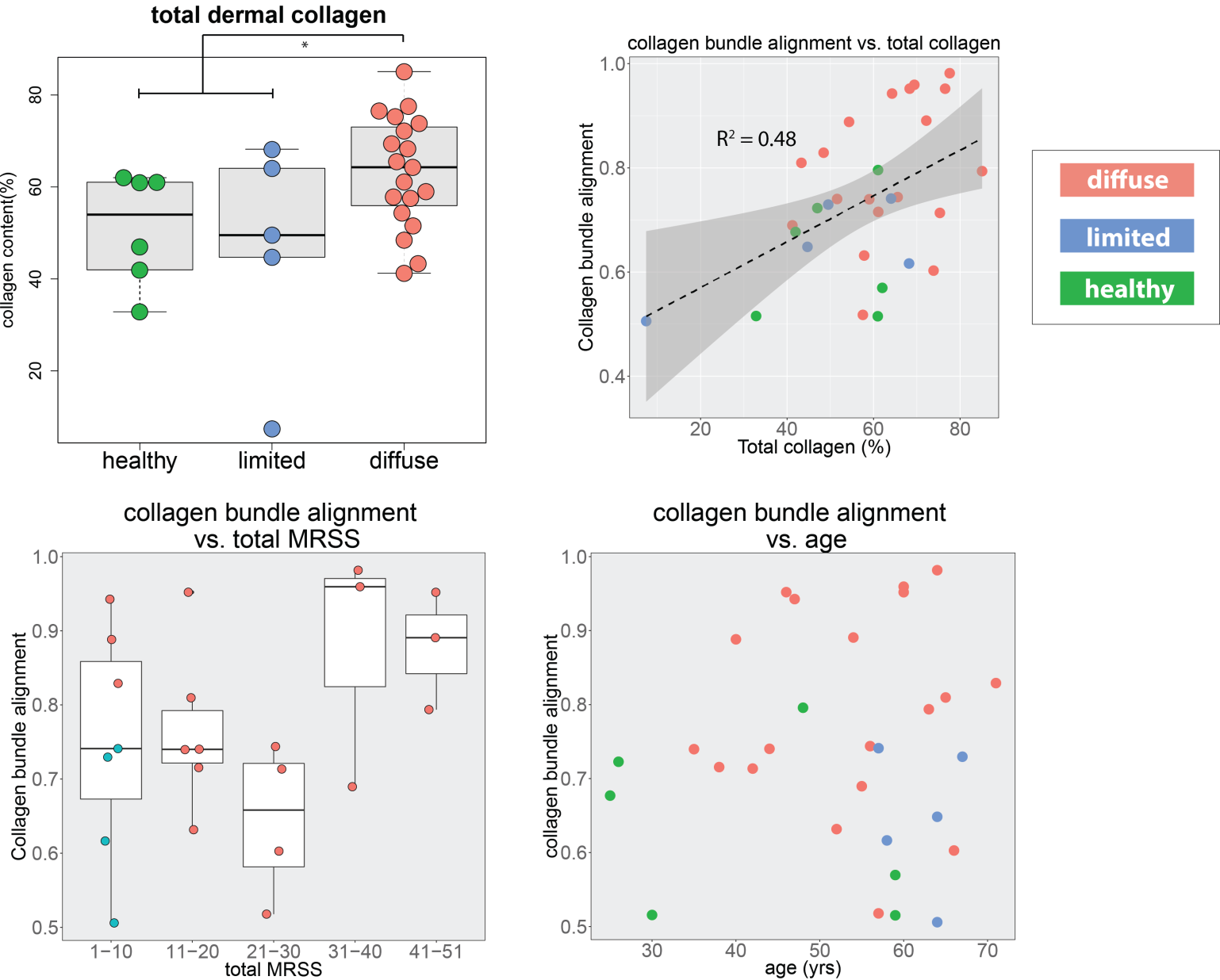


Total dermal collagen (upper left) in healthy (n-6), lcSSc (n=5) and dcSSc (n=19) samples. (Boxplot represent first quartile, median, and third quartile. Whiskers show min to max. *, p < 0.05, ANOVA, error bars are SEM). Correlation of dermal collagen bundle alignment to total dermal collagen (upper right) (R^2^ = 0.48, p = 0.006, pearson, shaded area represents 95% CI for regression), and no correlations were found to clinical parameters such as MRSS, and age (lower graphs).

**Figure C.** aSMA IHC staining of human dcSSc skin


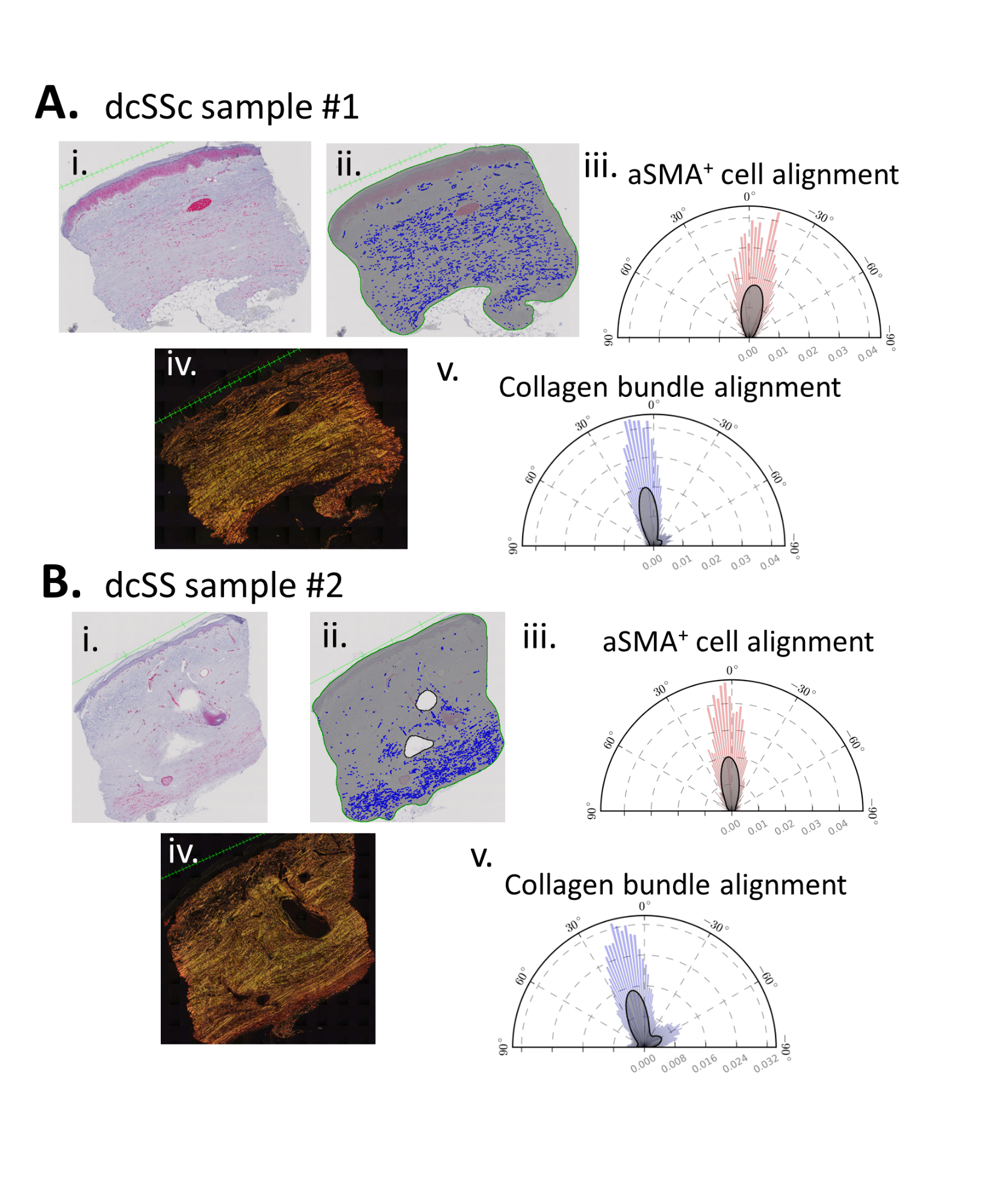


Alpha-smooth muscle actin(aSMA) IHC staining from 2 independent dcSSc skin samples (**A** and **B**). (i), whole-slide image of aSMA stained tissue section. (ii), Masking and detection of aSMA+ fibroblastic cells. (iii), Histogram visualization of the alignment of aSMA+ fibroblasts, distribution of alignment angles relative to epidermis. (iv), corresponding section, staining of collagen by picosirius red (PSR) and imaged under polarized light microscopy. (v), Histogram visualization of collagen bundle alignment, distribution of fiber bundle angles relative to the epidermis. Location of epidermis is indicated by dashed green line.

**Figure D.** Skin biopsy sections from subcutaneous bleomycin-induced mouse model of SSc during bleomycin treatment and recovery timepoints


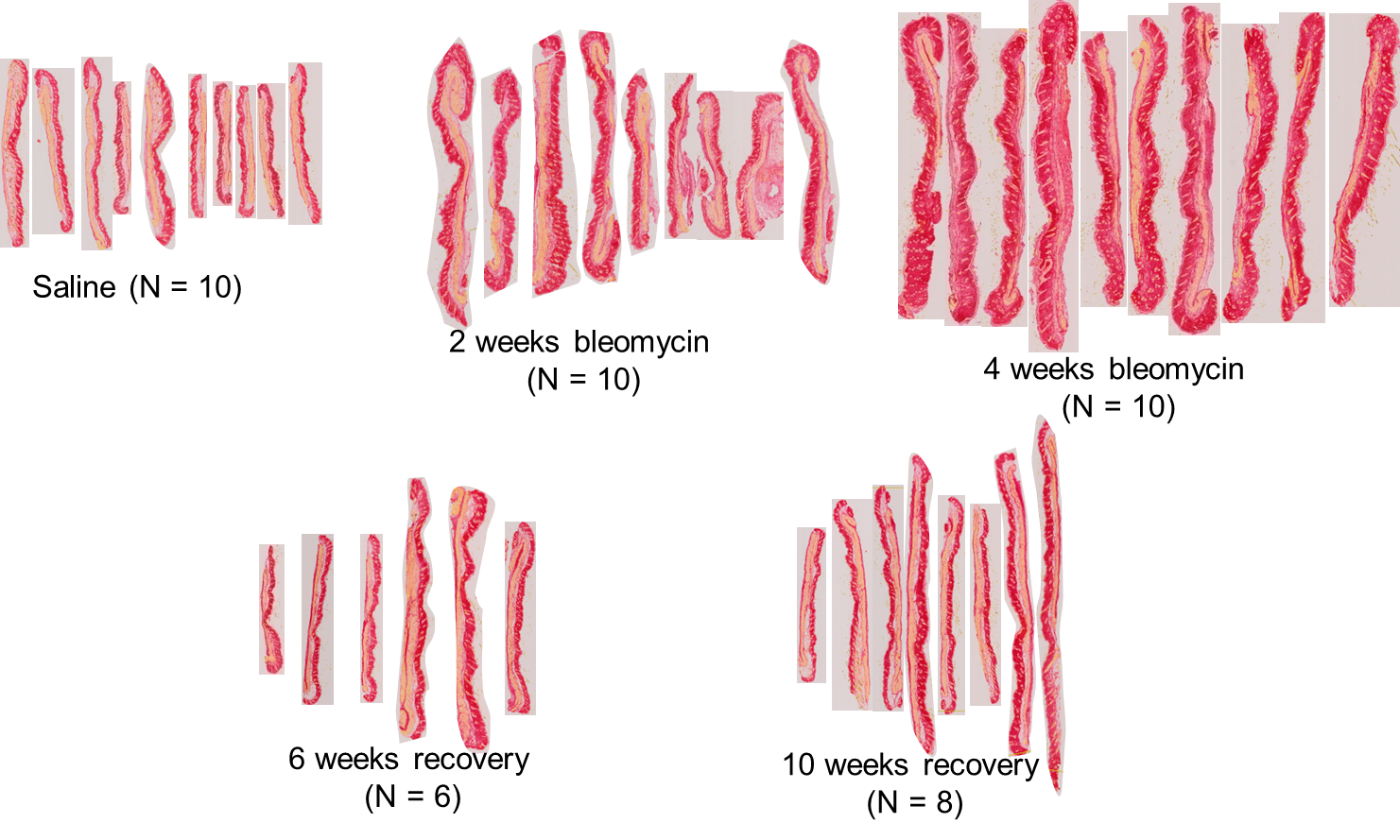
 Full thickness skin biopsy sections stained for collagen with PSR (imaged at 0.5X) from 8 week old female C57Bl/6 mice that received daily injections of bleomycin (0.1U/mL, 100uL) into the back skin. The study design included the following groups: saline, 2 weeks or 4 weeks bleomycin treatment, and 2 groups in which animals were dosed with bleomycin for 4 weeks and then allowed to recover for an additional 6 or 10 weeks to assess recovery. Peak fibrosis occurred by 4 weeks of bleomcyin treatment.

**Figure E.** Dermal collagen bundle alignment in bleomycin-induced fibrosis in mice reflects a distinct feature that is not correlated with dermis thickness or total dermal collagen


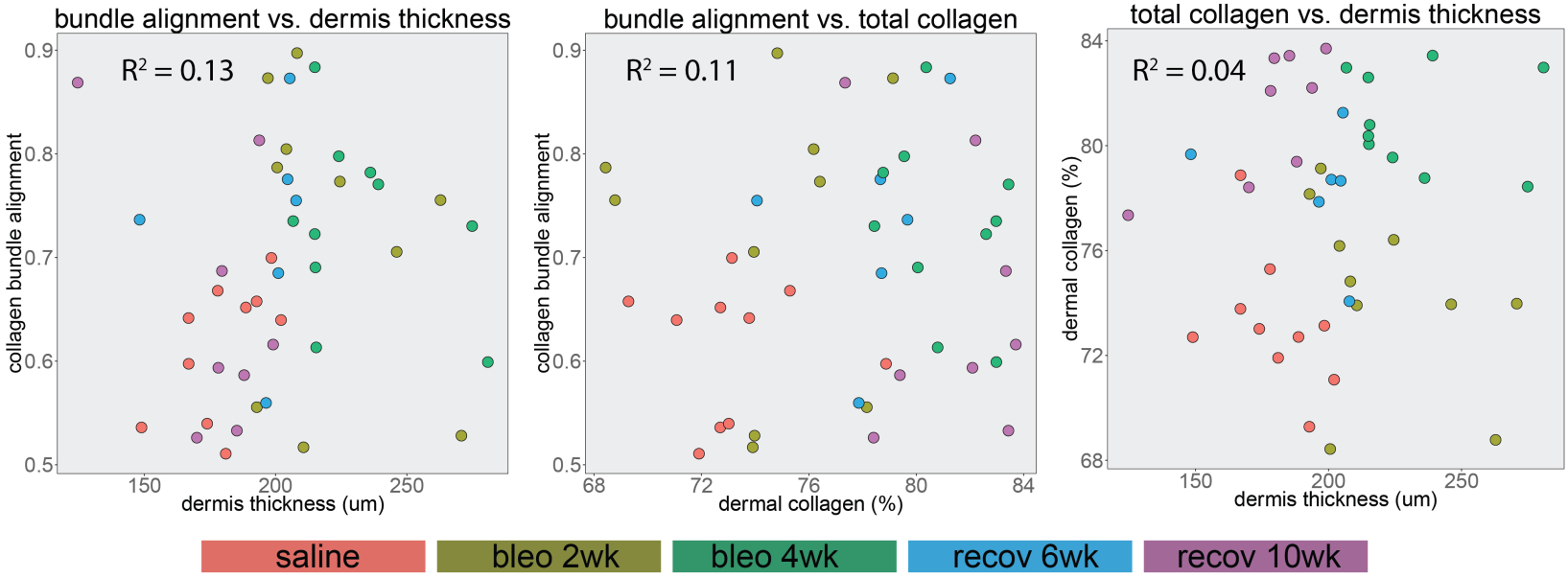


Fibrosis was induced by bleomycin treatment in the cohorts described in Figure 2. Correlations of all three parameters (dermis thickness, collagen bundle alignment, and total collagen) with one another. Bundle alignment vs. dermis thickness, R^2^ = 0.13, p = 0.39, pearson. Bundle alignment vs. total collagen, R^2^ = 0.11, p = 0.46, pearson. Total collagen vs. dermis thickness, R^2^ = 0.04, p = 0.80, pearson. No significant correlations were found between any of these parameters, suggesting that each informs a distinct feature of bleomycin induced fibrosis.

**Figure F.** Core set of concertedly upregulated genes associated with bleomycin-induced increased dermal collagen bundle alignment


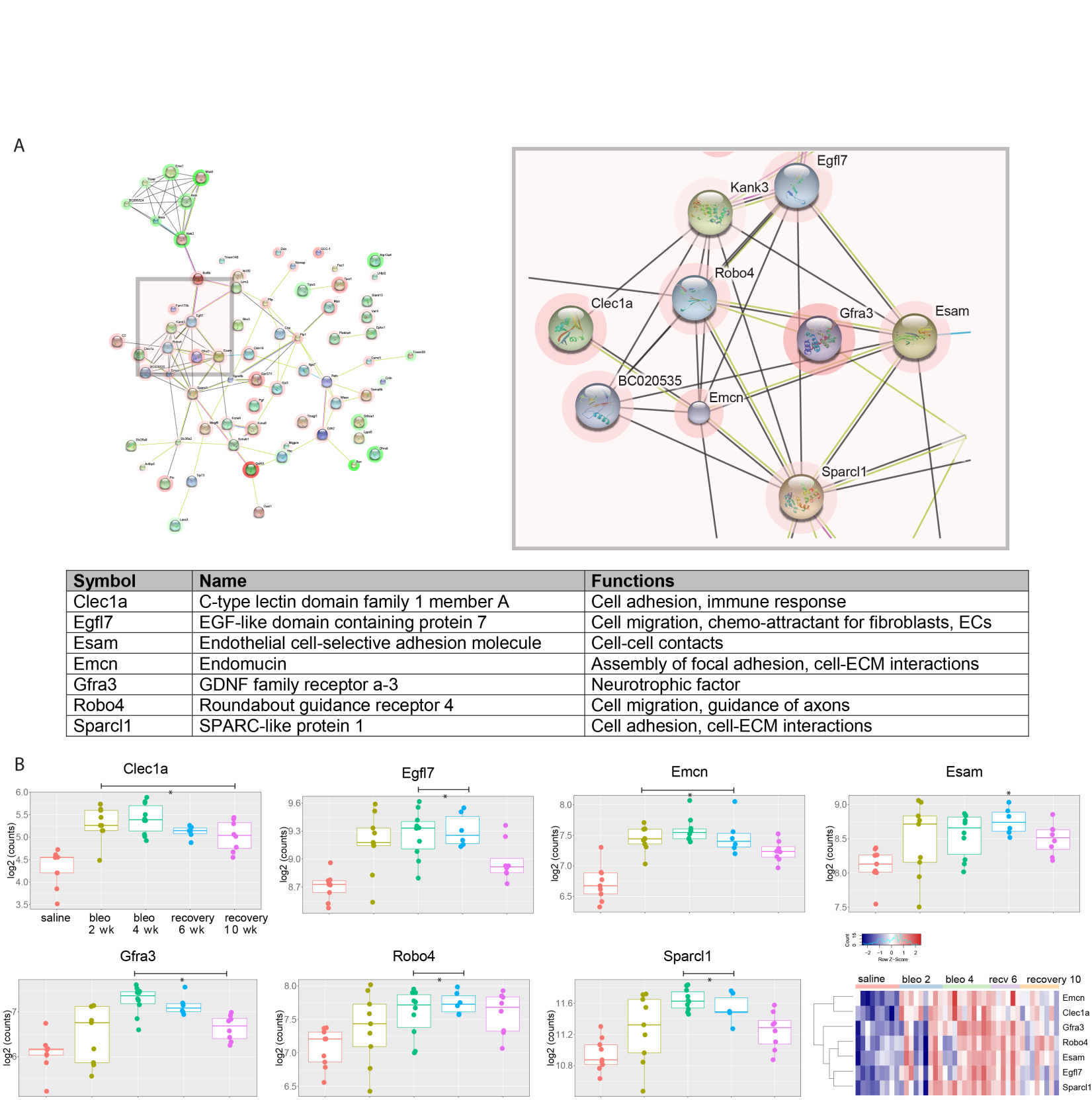


**(A)** Pathway analysis of the 81 unique collagen bundle alignment-associated genes using STRINGdb identified a core set of 7 genes that are concertedly upregulated (inset, shown on right). Common to these genes are their reported functions in cell adhesion and migration. **(B)** Expressed levels of each gene, across different sample groups analyzed, and heatmap representation of this set of genes, showing concerted upregulation in bleomycin and recovery groups compared to saline. (*, significance vs saline, using DESeq2. See methods for DEG criteria)

**Figure G.** Set of concertedly upregulated genes are positively correlated with collagen bundle alignment


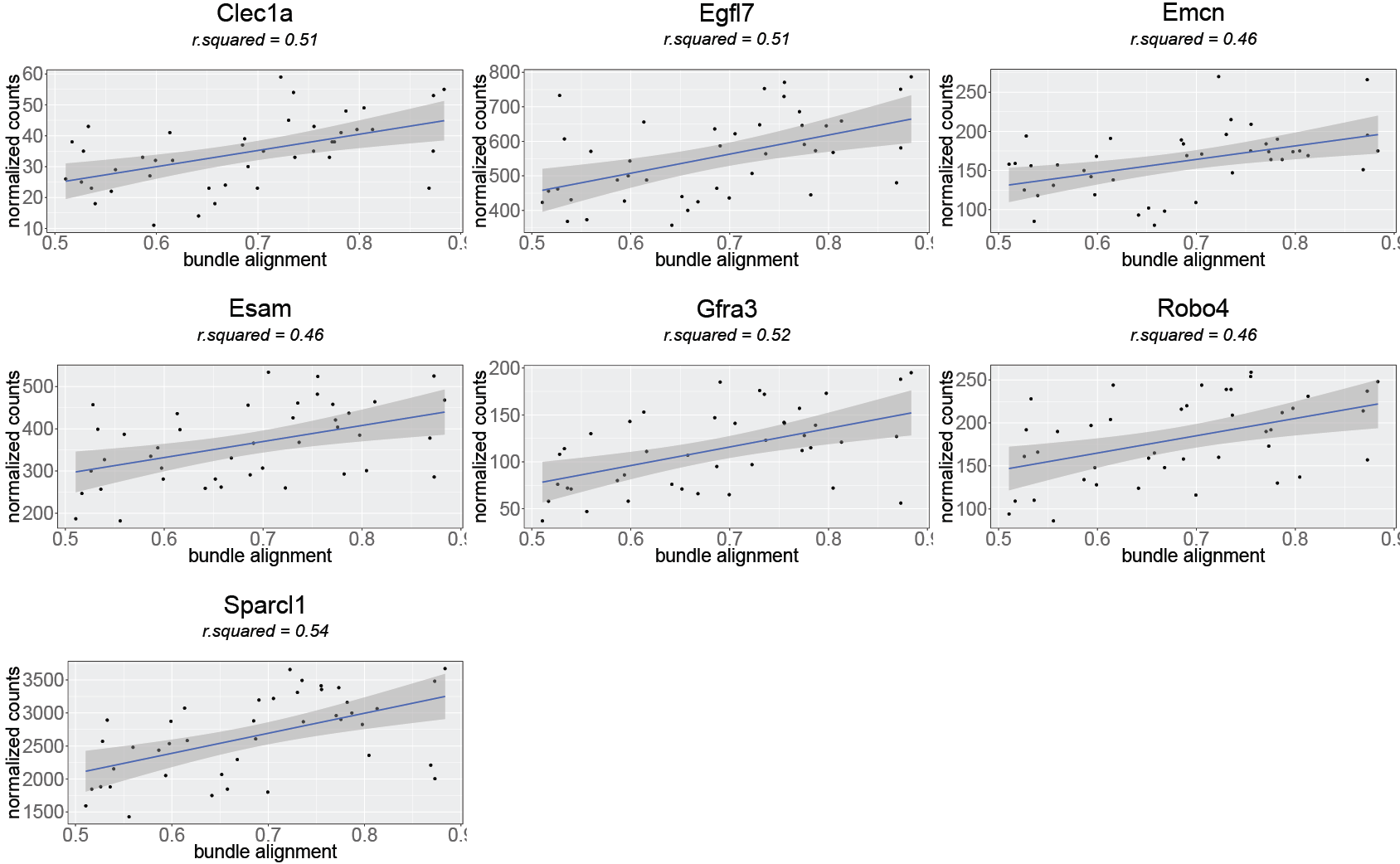


Correlation of expression level of each indicated gene with collagen bundle alignment, across all samples analyzed (N = 44). Pearson correlation coefficients are shown for each gene. **Figure H.** Human primary dermal fibroblasts show an enhanced migratory phenotype when cultured on substrates with collagen-adsorbed aligned ECM nanofibers


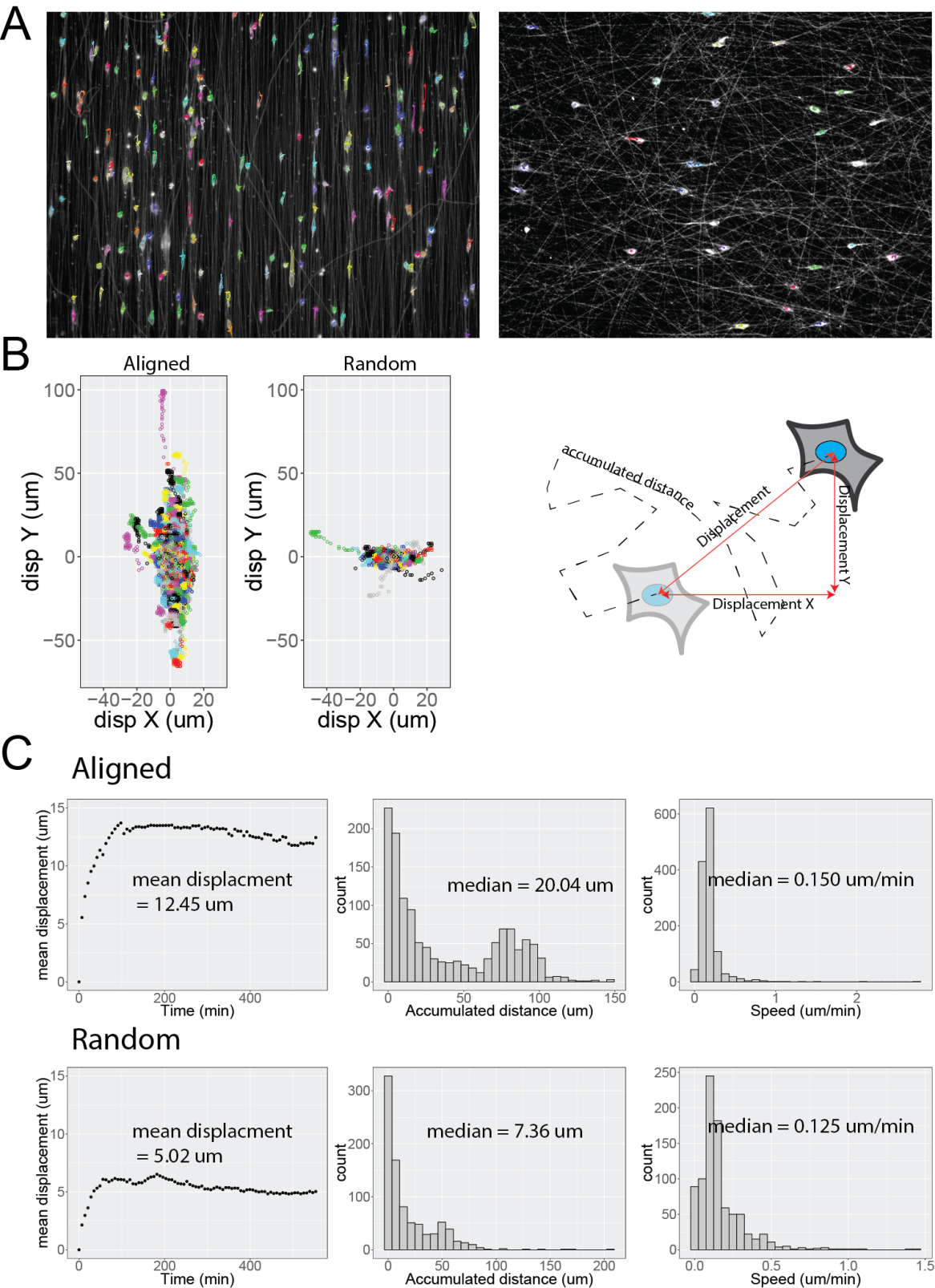


Assay of fibroblast migration by time lapse imaging. Cells were loaded with CellTracker Red CMTPX dye, and imaged overnight at 7 minute intervals on a PerkinElmer Operetta microplate imager overnight. Representative images showing tracked cells in psedocolor, at initial time point in aligned nanofiber culture conditions (left), and randomly-oriented nanofiber culture conditions (right) (**B)** Migration tracks shown as displacement in Y and X directions (disp Y and disp X) of dermal fibroblasts on aligned nanofibers (left), and randomly-oriented nanofibers (right) with adsorbed type I collagen, over the duration of the experiment. Individual cells are represented by a separate color track. Approximately 1300 cells were tracked per condition. **C)** Migration parameters (mean displacement, accumulated distance, and speed) quantified for cells cultured on aligned (top) vs. random (bottom) nanofibers. Mean displacement is approximately 2.4X farther on aligned group, and total distance migrated is 2.8X longer on aligned group. **Figure I.** Efficiency of siRNA inhibition of *Arhgdib*

qPCR of *Arhgdib* mRNA levels, and efficiency of siRNA knockdown. Cells were transfected with silencer select siRNA (ThermoFisher, Waltham, MA) against *Arhgdib* using 3 independent siRNAs, or a negative control siRNA.

**Figure J.** Collagen bundle alignment in scleromatous graft versus host disease (scl-GVHD) mouse model recapitulates collagen bundle alignment features of human dcSSc


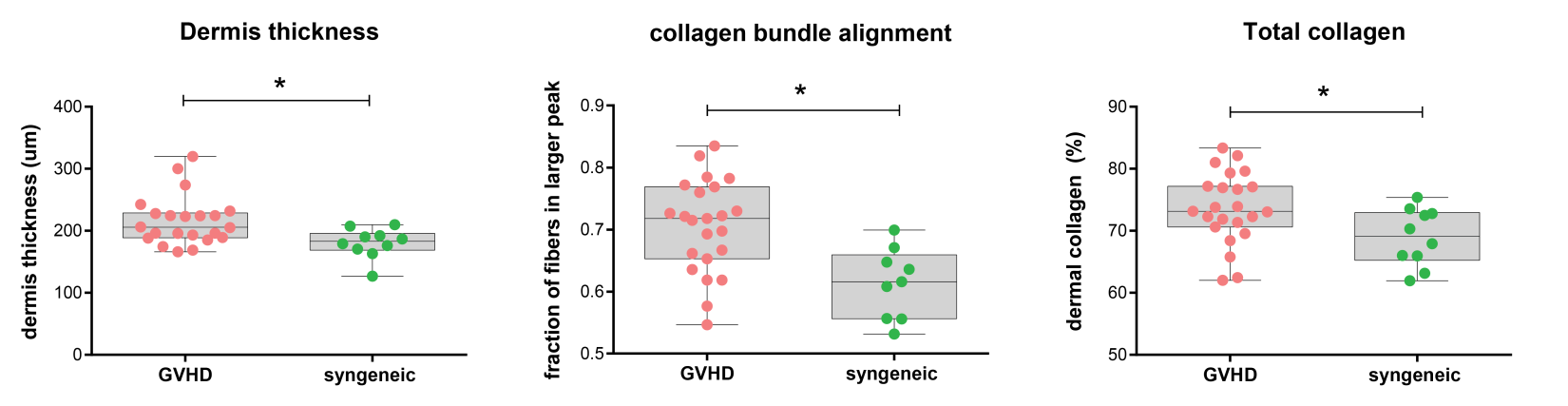
Changes to dermal architecture in the scl-GVHD mouse model of SSc, wherein 2x10^6^ splenocytes were transferred from donor B10.D2 strain (Jackson Laboratory) to a recipient C.129S6(B6)-Rag2^tm1Fwa^N12 strain (Taconic) to induce disease. Splenocytes were transferred from a wildtype BALB/c donor strain for syngeneic controls. Back skin was taken by biopsy, at the same location for each animal, and collagen was analyzed. Collagen bundle alignment, dermis thickness, and total collagen were all increased in scl-GVHD skin as compared to syngeneic control skin. (Boxplot represent first quartile, median, and third quartile. Whiskers show min to max. *, p < 0.05, t-test)
